# Human Properdin Modulates Macrophage: *Mycobacterium bovis* BCG Interaction *via* Thrombospondin Repeats 4 and 5

**DOI:** 10.3389/fimmu.2018.00533

**Published:** 2018-05-08

**Authors:** Maha Ahmed Al-Mozaini, Anthony G. Tsolaki, Munirah Abdul-Aziz, Suhair M. Abozaid, Mohammed N. Al-Ahdal, Ansar A. Pathan, Valarmathy Murugaiah, Evgeny M. Makarov, Anuvinder Kaur, Robert B. Sim, Uday Kishore, Lubna Kouser

**Affiliations:** ^1^College of Health and Life Sciences, Brunel University London, London, United Kingdom; ^2^Department of Infection and Immunity, King Faisal Specialist Hospital and Research Centre, Riyadh, Saudi Arabia; ^3^Department of Biochemistry, Oxford University, Oxford, United Kingdom

**Keywords:** complement, cytokine, properdin, macrophage, *Mycobacterium tuberculosis*, *Mycobacterium bovis* BCG, phagocytosis, thrombospondin repeats

## Abstract

*Mycobacterium tuberculosis* can proficiently enter macrophages and diminish complement activation on its cell surface. Within macrophages, the mycobacterium can suppress macrophage apoptosis and survive within the intracellular environment. Previously, we have shown that complement regulatory proteins such as factor H may interfere with pathogen–macrophage interactions during tuberculosis infection. In this study, we show that *Mycobacterium bovis* BCG binds properdin, an upregulator of the complement alternative pathway. TSR4+5, a recombinant form of thrombospondin repeats 4 and 5 of human properdin expressed in tandem, which is an inhibitor of the alternative pathway, was also able to bind to *M. bovis* BCG. Properdin and TSR4+5 were found to inhibit uptake of *M. bovis* BCG by THP-1 macrophage cells in a dose-dependent manner. Quantitative real-time PCR revealed elevated pro-inflammatory responses (TNF-α, IL-1β, and IL-6) in the presence of properdin or TSR4+5, which gradually decreased over 6 h. Correspondingly, anti-inflammatory responses (IL-10 and TGF-β) showed suppressed levels of expression in the presence of properdin, which gradually increased over 6 h. Multiplex cytokine array analysis also revealed that properdin and TSR4+5 significantly enhanced the pro-inflammatory response (TNF-α, IL-1β, and IL-1α) at 24 h, which declined at 48 h, whereas the anti-inflammatory response (IL-10) was suppressed. Our results suggest that properdin may interfere with mycobacterial entry into macrophages via TSR4 and TSR5, particularly during the initial stages of infection, thus affecting the extracellular survival of the pathogen. This study offers novel insights into the non-complement related functions of properdin during host–pathogen interactions in tuberculosis.

## Introduction

Properdin is an upregulator of the alternative pathway of complement activation. In one of the three pathways of the complement system, the alternative pathway, the activation of the major complement opsonin, C3, is driven by a complex serine protease, C3bBb, also called the C3 convertase, which is bound to the surface of the complement-activating target. To form C3bBb, factor B associates with C3b in the presence of Mg^2+^ and factor D, a serine protease, which cleaves factor B into Bb and Ba fragments producing a C3 convertase C3bBb (1). This complex, which has a half-life of 90 s, is stabilized by the binding of properdin, which increases the half-life by 5- to 10-fold (2). Furthermore, C3b molecules are generated by C3bBb and deposited near to the surface-bound convertase leading to the opsonization of the target and formation of C5 convertase, producing C5a and C5b, leading on to the lytic pathway and cell lysis (3).

The monomer of human properdin (53 kDa) has a flexible rod-like structure with a length of 26 nm and a diameter of 2.5 nm, composed of seven thrombospondin type I repeats (TSR). Each TSR is of about 60 amino acids, typically containing six conserved cysteine residues: these occur in TSR1–TSR6 (4–6), but the N-terminal domain, TSR0, is truncated. TSR4 is crucial for binding to C3bBb and TSR5 for binding to C3bBb, suggesting that both TSRs may be important for stabilizing the C3 convertase complex (5). Recently, TSR4+5, expressed as a double domain, has been shown to bind to properdin ligands such as C3b and inhibit the alternative complement pathway (7). These studies demonstrate the important role that these TSRs may play in the alternative pathway and in their interaction with pathogens.

Properdin circulates in plasma at a concentration of about 4–25 µg/ml existing as cyclic oligomers, dimer, trimer, and tetramer in a ratio of 26:54:20 (4). The dissociation and reassociation of properdin upon denaturation–renaturation cycles stimulated by guanidine or low pH indicates properdin ratio stability in solution (8). The interaction between properdin monomers involves the N-terminal end of one monomer and the C-terminal end of another (9). Properdin can also bind to microbial surfaces of several pathogens, including *Neisseria gonorrhoeae* (10), *Salmonella typhimurium* lipopolysaccharide (LPS), *Neisseria meningitidis* lipooligosaccharide (11), and *Chlamydia pneumoniae* (12). Binding of properdin to microbial surfaces results in the recruitment of fluid phase C3b, inducing assembly of C3 convertase C3bBb and causing further deposition of C3b on the pathogen surface (13–15), subsequently generating a C5 convertase, MAC formation, and cell lysis. At 10 µg/ml, recombinant properdin enhanced complement deposition on *N. meningitidis* and *S. pneumoniae* and dramatically enhanced serum lysis of these bacteria, and in the mouse model, significantly reduced bacteremia and increased survival rates (16).

Although *Mycobacterium tuberculosis* and its close-relative *Mycobacterium bovis* have significant interaction with components of the innate immune system, e.g., toll-like receptors, complement, surfactant proteins SP-A and SP-D (17), the initial stages of tuberculosis pathogenesis remain poorly understood. Many bacteria have evolved mechanisms to evade immune responses: by inhibiting complement activation by proteolytic cleavage of complement proteins, having their own complement inhibitors (18), or binding complement regulatory proteins like factor H (15, 19).

*Mycobacterium tuberculosis* is a highly specialized intracellular pathogen and may exploit complement proteins to enhance its uptake by macrophages. Although it has been shown that *M. tuberculosis* can activate all three pathways of the complement system (20, 21), it is unclear how the pathogen uses complement proteins in tuberculosis pathogenesis. *M. tuberculosis* has been shown to bind to complement receptors (CR) CR1, CR3, and CR4 and gain entry into macrophages (22–24). There is also evidence that enhanced phagocytosis of *M. tuberculosis* by human alveolar and monocyte-derived macrophages results from C3 opsonization (24). The ability of *M. tuberculosis* to bind to CR3 non-opsonically has also been shown which may be important for bacterial invasion when complement is sparse, for example, in the lung (25). Properdin has recently been considered as a pattern recognition receptor (PRR) on its own, i.e., binding to recognition patterns without need for prior deposition of C3b or C3bBb (26–28). Therefore, we investigated the role of properdin in tuberculosis pathogenesis, by using the model organism *M. bovis* BCG.

Here, we show, for the first time, that properdin and recombinant form of TSR4+5 expressed as a two-module protein binds to *M. bovis* BCG, demonstrating its role as a soluble PRR. Properdin and TSR4+5 were found to inhibit the uptake of *M. bovis* BCG by macrophages during phagocytosis, altering the pro- and anti-inflammatory cytokine response, and thus, possibly shaping the adaptive immune response in tuberculosis pathogenesis.

## Materials and Methods

### Purification of Native Properdin

The affinity columns, IgG Sepharose and anti-properdin monoclonal antibody Sepharose, were prepared as described previously (7). The IgG-Sepharose column was prepared from human non-immune IgG (~26 mg IgG/ml of Sepharose) coupled to CNBr-activated Sepharose (GE Healthcare, UK). For preparation of the anti-human properdin column, CNBr-activated Sepharose (GE Healthcare Life Sciences, UK) was used to couple to anti-properdin mouse monoclonal antibody (2 mg/ml). One liter of human plasma (TCS Biosciences) containing 5 mM EDTA was filtered through Whatman filter paper before applying to IgG Sepharose to deplete C1q (which would otherwise have bound to the IgG on the anti-properdin Sepharose). The column was washed with three bed volume of HEPES buffer (10 mM HEPES, 140 mM NaCl, 0.5 mM EDTA, and pH 7.4). Plasma was then applied to the monoclonal anti-properdin column and washed with the same HEPES buffer. Bound properdin was eluted with 3 M MgCl_2_ and the peak fractions were dialyzed against HEPES buffer overnight at 4°C. Contaminants were further removed by applying the pooled protein fractions to a HiTrap Q FF-Sepharose (GE Healthcare) ion-exchange column, followed by washing the column with three bed volumes of 50 mM Tris–HCl, pH 7.5, 50 mM NaCl, and 5 mM EDTA. Properdin did not bind to the Q Sepharose column and appeared in the flow-through free from contaminants as demonstrated by SDS-PAGE.

For the size exclusion chromatography analysis, 50 µl of the proteins at the concentrations varying from 0.3 mg/mL to 1.0 mg/mL were applied to a TSKgel G2000SWXL, 5 µm, 7.8 × 300 mm column (Tosoh Bioscience). The column was equilibrated with buffer containing 50 mM sodium phosphate, pH7.0 and 300 mM NaCl at the flow rate 0.3 ml/min using SCL-10Avp HPLC system (Shimadzu). The absorbance was detected at 230 and 280 nm. The Bio-Rad Gel Filtration Standard (Cat # 151-1901) were used for the protein molecular weight calibration of the column.

### Expression and Purification of TSR4+5

The recombinant maltose-binding protein (MBP) fusion proteins MBP-TSR4+5, MBP-TSR4, or MBP-TSR5 were expressed in *Escherichia coli* as described previously (7, 29). The *E. coli* BL21 bacterial cells (Life Technologies) were grown in 1 L of Luria–Bertani medium with 100 µg/ml ampicillin, shaking at 37°C until an optical density at 600 nm (OD_600_) of between 0.6 and 0.8 was reached. Protein expression was then induced in the bacterial cell culture with 0.4 mM isopropyl β-d-1-thiogalactopyranoside (IPTG) (Sigma-Aldrich) for 3 h shaking at 37°C. The cells were then pelleted at 4,500 rpm, 4°C for 10 min, lysed using 50 ml lysis buffer [20 mM Tris–HCl, pH 8.0, 0.5 M NaCl, 1 mM EDTA, 0.25% v/v Tween 20, 5% v/v glycerol, 100 µg/ml lysozyme (Sigma-Aldrich), and 0.1 mM phenylmethanesulfonyl fluoride (Sigma-Aldrich)], and incubated for 1 h at 4°C on a rotary shaker. The cell lysate was then sonicated using a Soniprep 150 (MSE, London, UK) at 60 Hz for 30 s with an interval of 2 min (12 cycles) and then centrifuged at 13,000 rpm for 15 min at 4°C. The supernatant was diluted five-fold with buffer A (20 mM Tris–HCl, pH 8.0, 100 mM NaCl, 1 mM EDTA, and 0.25% v/v Tween 20) and passed through an amylose resin column (25 ml bed) (New England Biolabs) that was equilibrated in buffer A. The affinity column was washed with buffer A without Tween 20 and with 1 M NaCl, 20 mM Tris–HCl, pH 8.0, 1 mM EDTA, followed by buffer B (20 mM Tris–HCl, pH 8.0, 100 mM NaCl, 1 mM EDTA). The MBP-TSR4+5 fusion protein was eluted with 100 ml of buffer B containing 10 mM maltose (Sigma-Aldrich) (affinity elution buffer). Trace contaminants were further removed by applying the fusion protein to a DEAE Sepharose column. Thus, the affinity purified fusion protein in affinity elution buffer was applied to the ion-exchange (5 ml bed) column and washed with three column volumes of low salt buffer containing 50 mM Tris–HCl, pH 7.5, 100 mM NaCl, 5 mM EDTA, at pH 7.5. After extensive washing with low salt buffer, the fusion protein eluted at 0.2 M NaCl using a NaCl gradient (50 mM to 1 M). The peak elutions were then passed through Pierce™ High Capacity Endotoxin Removal Resin (Qiagen) to remove LPS. Endotoxin levels were determined using the QCL-1000 Limulus amebocyte lysate system (Lonza), and the assay was linear over a range of 0.1–1.0 EU/ml (10 EU = 1 ng of endotoxin). The endotoxin levels were less than 4 pg/µg of the MBP-TSR4+5.

### Mycobacterial Cell Culture

*Mycobacterium bovis* BCG (Pasteur strain) were grown in liquid culture using Middlebrook 7H9 media (Sigma-Aldrich), supplemented with 0.2% (v/v) glycerol, 0.05% (v/v) Tween-80, and 10% (v/v) albumin dextrose catalase (ADC) (BD BBL, Becton Dickinson). Green fluorescent protein (GFP)-expressing *M. bovis BCG* (Danish Strain 1331) containing the pGFPHYG2 plasmid was a kind gift from B. Robertson, Imperial College, London, UK. GFP-*M. bovis* BCG was grown in the above conditions/media but with the addition of 50 µg/ml of hygromycin to maintain the plasmid. Cultures were incubated at 37°C with agitation (~120 rpm) for 7–10 days until the bacteria had reached the exponential growth phase at OD_600nm_ = 0.60–1.00.

### Assay of Human Properdin and TSR4+5 Binding to Mycobacteria

*Mycobacterium bovis* BCG, harvested and washed in PBS, was adjusted to a concentration of 1.25 × 10^9^ cells/ml in PBS (OD_600_ = 1 equates to approximately 1 × 10^9^ cell/ml). Then 200 µl of bacterial suspension was dispensed into individual microtiter wells of a 96-well plate (Maxisorp™, NUNC). Plates were incubated at 4°C overnight and washed with buffer 1 [10 mM HEPES pH 7.5, 140 mM NaCl, 0.5 mM EDTA, and 100 µg/ml hen ovalbumin (Sigma-Aldrich)]. Wells were blocked for 2 h at 37°C with buffer 1 + 10% (w/v) Marvel Dried Milk powder.

Human properdin (up to 50 µg/ml) or TSR4+5 (up to 30 µg/ml) were added, in two-fold serial dilutions (100 µl/well) in buffer 1 and incubated for 2 h at 37°C. Individual TSR4 and TSR5 proteins, MBP and BSA were used as negative controls. Microtiter wells were washed three times with buffer 1. Mouse anti-properdin monoclonal antibody (1.19 mg/ml) diluted 1/2,500 in buffer 1 (29) was added to the wells containing properdin. Mouse anti-MBP monoclonal antibody (Sigma-Aldrich) was added to wells containing TSR4+5, TSR4 and TSR5, diluted 1/5,000 in buffer 1, and incubated for 1 h at 37°C. For the BSA negative control, mouse anti-BSA monoclonal antibody (Sigma-Aldrich) was used (1/5,000 dilution). Plates were washed an additional three times in buffer 1 and then incubated with goat anti-mouse IgG-horseradish peroxidase conjugate (Sigma-Aldrich), diluted 1/5,000 in buffer 1. The substrate *p*-nitrophenol phosphate (Sigma-Aldrich) was then added to each well, and the plates read at 405 nm.

### Fluorescence Microscopy for TSR4+5 Binding to Mycobacteria

*Mycobacterium bovis* BCG bacteria (approximately 10^6^ cells) were spotted on poly-l-lysine coated microscope slides (Sigma-Aldrich) and incubated at 37°C for cells to adhere. After washing three times with PBS, bacterial cells were then fixed with 4% paraformaldehyde for 5 min. Slides were washed three times with PBS and then incubated at 37°C for 1 h with 0, 1, or 10 μg/ml of TSR4+5, or 10 µg/ml of BSA (negative control) in buffer 1. Slides were washed three times with PBS, and then the primary monoclonal antibody (mouse anti-MBP) added at 1/500 dilution and incubated for 1 h at room temperature. After washing three times with PBS, goat anti-mouse conjugated with AlexaFluor488 (1/500 dilution) was added as the secondary antibody and incubated for 1 h at room temperature. Slides were then washed three times with PBS and mounted with antifade (Citifluor AF3) PBS solution and viewed using a LeicaDM4000 Fluorescence microscope. Images were processed using Image J.^1^

### Phagocytosis Assay

THP-1 macrophage cells were cultured in RPMI-1640 (Gibco) (RPMI) containing 10% (v/v) fetal bovine serum (FBS) (Sigma-Aldrich), 2 mM l-glutamine (Sigma-Aldrich), 100 U/ml penicillin (Sigma-Aldrich), 100 µg/ml streptomycin (Sigma-Aldrich), and 1 mM sodium pyruvate (Sigma-Aldrich) and left to grow in 5% CO_2_ at 37°C for approximately 3 days before passaging. Cells were resuspended in RPMI and adjusted to 1 × 10^6^ cells/well (in 1.8 ml) in a 24-well plate. To induce adherence onto the wells, THP-1 cells were treated with 50 ng/ml of phorbol 12-myristate 13-acetate (PMA) (Sigma-Aldrich) into RPMI-1640 without FBS, penicillin or streptomycin and left to settle for at least 30 min before adding 200 µl of bacterial culture (1 × 10^9^ bacteria/ml).

*M. bovis* BCG bacteria were pelleted at mid-exponential phase, at an OD_600nm_ = 0.6–1.0 by centrifugation at 1,000 × *g* for 10 min at 4°C. The mycobacterial pellet was resuspended in the buffer 1. This mycobacterial culture was then separated into different microfuge tubes and treated with varying concentrations of properdin (2 or 20 µg/ml) or MBP-TSR4+5 (1 or 5 µg/ml). Control samples were left untreated, and all were incubated for 2 h at 37°C for binding to occur. The mycobacterial suspension was washed once in growth medium before resuspending in RPMI medium without FBS, penicillin, or streptomycin. 200 µl of the mycobacterial suspension was added to each well of THP-1 cells. Mycobacterial concentration was adjusted to give approximate multiplicity of infection (MOI) ratio of 10:1.

Plates were gently swirled and incubated at 37°C, 5% CO_2_ for up to 48 h to allow mycobacterial uptake. THP-1 cells were sampled at 15, 30, and 45 min and 1, 2, and 6 h. Supernatants were collected after 24 and 48 h of incubation for multiplex analysis. Plates were washed three times with PBS to remove extracellular bacteria. THP-1 cells were then lifted by adding 1 ml of 0.25% trypsin to the wells and incubated for 10 min at 37°C, 5% CO_2_. THP-1 cells were collected by centrifugation at 1,000 × *g* for 10 min at 4°C.

To recover and count the ingested mycobacteria, THP-1 cells were lysed by resuspending the cell pellets in 1 ml of sterile water, followed by a series of vortex mixing for 10 min at room temperature. 24-well plates containing 2 ml Middlebrook 7H10 agar with 10% Oleic Acid+ADC (OADC) (BD, BBL, Becton Dickinson) were prepared. Four serial 1/10 dilutions were made, and 10 µl of the concentrated mycobacterial suspension and diluted suspension from each time point was spotted onto the 7H10 agar wells. The 24-well plates were secured with parafilm and wrapped in aluminum foil, inverted and incubated at 37°C for 10–14 days. Wells were photographed, and the colony-forming unit (CFU) count determined. The same procedure was used to quantify the initial input number of bacteria incubated with THP-1 cells.

### Fluorescence Microscopy for Phagocytosis Assay

THP-1 cells were cultured as described above and seeded at 1 × 10^5^ cells per 13 mm coverslip and differentiated with PMA as described above. GFP-expressing *M. bovis* BCG was incubated with 0, 1, 10 µg/ml of TSR4+5 for 2 h at 37°C in buffer 1. Cells were also incubated with 10 µg/ml of BSA as a negative control. Cells were washed twice in PBS and then resuspended in plain RPMI media. 1 × 10^6^ GFP-*M. bovis* BCG was added to the THP-1 cells (MOI of 10:1) and incubated for phagocytosis for 2 h at 37°C. THP-1 cells were then washed three times in PBS to remove extracellular bacteria and then fixed in 4% paraformaldehyde for 5 min. After washing three times in PBS, THP-1 cells were incubated with 2 µg/ml of AlexaFluor546-conjugated wheat germ agglutinin (Invitrogen) to reveal the plasma membrane. Cells were then washed three times and mounted using Vectashield antifade with DAPI (Vector Labs) to reveal nucleus. Slides were observed under a Leica DM4000 fluorescence microscope at 40× magnification. Images were processed using Image J (see text footnote 1).

### Quantitative Real-Time PCR (qPCR) Analysis of mRNA Expression of Cytokines

THP-1 cell pellets were collected from each time point as described above. RNA extraction was performed using the GenElute Mammalian Total RNA Purification Kit (Sigma-Aldrich) according to the manufacturer’s protocol. Samples were then treated with DNase I (Sigma-Aldrich) to remove any contaminating DNA according to the manufacturer’s protocol. The amount of RNA was measured using the NanoDrop 2000/2000c spectrophotometer (Thermo Fisher Scientific) at 260 nm, and the ratio of absorbance at 260 and 280 nm was used to assess the purity of the RNA. Complementary DNA (cDNA) was synthesized using High Capacity RNA to cDNA Kit (Applied Biosystems, UK) according to the manufacturer’s protocol. Primer sequences (Table 1) were designed and analyzed for specificity using the nucleotide Basic Local Alignment Search Tool and Primer-BLAST.^2^

**Table 1:** Primers used for quantitative real-time PCR.

	Forward primer	Reverse primer
18S	5′-ATGGCCGTTCTTAGTTGGTG-3′	5′-CGCTGAGCCAGTCAGTGTAG-3′
IL-1β	5′-GGACAAGCTGAGGAAGATGC-3′	5′-TCGTTATCCCATGTGTCGAA-3′
IL-6	5′-GAAAGCAGCAAAGAGGCACT-3′	5′-TTTCACCAGGCAAGTCTCCT-3′
IL-10	5′-TTACCTGGAGGAGGTGATGC-3′	5′-GGCCTTGCTCTTGTTTTCAC-3′
IL-12	5′-AACTTGCAGCTGAAGCCATT-3′	5′-GACCTGAACGCAGAATGTCA-3′
TGF-β	5′-GTACCTGAACCCGTGTTGCT-3′	5′-GTATCGCCAGGAATTGTTGC-3′
TNF-α	5′-AGCCCATGTTGTAGCAAACC-3′	5′-TGAGGTACAGGCCCTCTGAT-3′

PCR was performed on all cDNA samples to assess the quality of the cDNA. The qPCR assays were performed for the expression of pro- and anti-inflammatory cytokines. The qPCR reaction consisted of 5 µl Power SYBR Green MasterMix (Applied Biosystems), 75 nM of forward and reverse primer, 500 ng template cDNA in a 10 µl final reaction volume. qPCR was performed in a 7900HT Fast Real-Time PCR System (Applied Biosystems). The initial steps were 2 min incubation at 50°C followed by 10 min incubation at 95°C, the template was then amplified for 40 cycles under these conditions: 15 s incubation at 95°C and 1 min incubation at 60°C. Samples were normalized using the expression of human 18S rRNA. Data were analyzed using the relative quantification (RQ) Manager Version 1.2.1 (Applied Biosystems). Cycle threshold (Ct) values for each cytokine target gene were calculated, and the relative expression of each cytokine target gene was calculated using the RQ value, using the formula: RQ = 2^−ΔΔCt^ for each cytokine target gene, and comparing relative expression with that of the 18S rRNA constitutive gene product. Assays were conducted twice in triplicate.

### Multiplex Analysis

Supernatants were collected from the phagocytosis assay at 24 and 48 h to determine the levels of secreted cytokines (IL-6, IL-10, IL-12p40, IL-12p70, IL-1α, IL-1β, TNF-α, IL-13, IL-15, IL-17A, IL-9, and TNF-β), chemokines (MCP-3, MDC, Eotaxin, Fractalkine, GRO, IL-8, IP-10, MCP-1, and MIP-1α), growth factors (IL-2, EGF, FGF-2, G-CSF, GM-CSF, IL-3, IL-4, IL-5, IL-7, and VEGF), and other related ligands and receptors (IFN-α2, IFN-ϒ, FLT-3L, IL-1RA, and sCD40L). MagPix Milliplex kit (EMD Millipore) was used to measure immune response following the manufacturer’s protocol. 25 µl of assay buffer was added to each well of a 96-well plate, followed by the addition of 25 µl of standard, controls or supernatants of cells treated with *M. bovis* BCG in the presence or absence of properdin and MBP-TSR4+5. 25 µl of magnetic beads coupled to analytes of interest was added in each well and incubated for 18 h at 4°C. The 96-well plate was washed with the assay buffer, and 25 µl of detection antibodies was incubated with the beads for 1 h at room temperature. 25 µl of streptavidin–phycoerythrin was then added to each well and incubated for 30 min at room temperature with shaking at 750 rpm. Following a washing step, 150 µl of sheath fluid was added to each well, and the plate was read using the Luminex Magpix instrument. Assays were conducted in duplicate.

### Statistical Analysis

Analysis of data for statistical significance was conducted using GraphPad Prism 6 for Windows (GraphPad Software, Inc.). Statistical analyses were made using two-way ANOVA for mRNA expression data and a one-way ANOVA for the multiplex data. *p* Values < 0.05 were considered statistically significant, unless otherwise stated (non-significant).

## Results

### Human Properdin and TSR4+5 Bind to Mycobacteria

**Figure 1:**
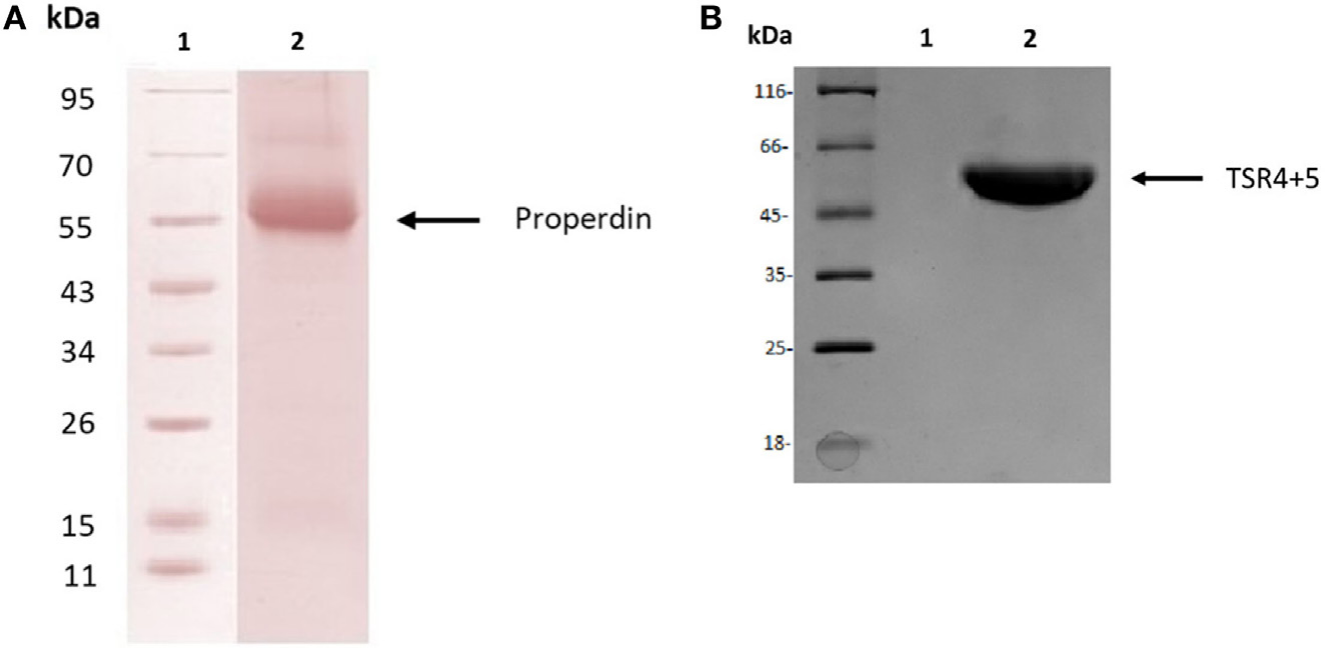
Purified human properdin and recombinant maltose-binding protein (MBP)-thrombospondin repeats (TSR) 4+5. **(A)** Properdin was purified from human plasma. Filtered plasma was applied to a non-immune IgG-Sepharose column, then to a mouse monoclonal anti-properdin Sepharose column; properdin was eluted with 3 M MgCl_2_. The eluted samples were dialyzed against HEPES buffer (10 mM HEPES, 140 mM NaCl, 0.5 mM EDTA, pH 7.4) overnight at 4°C. Contaminants were removed by applying the protein to a Q Sepharose column, and the product appears as a single band on SDS-PAGE and western blot at about 55 kDa. **(B)** MBP-TSR4+5 was purified *via* an amylose resin column, and the purified fusion protein also appears on SDS-PAGE as a band of about 55 kDa.

Human properdin was purified from human plasma. SDS-PAGE, followed by western blotting using antihuman properdin polyclonal antibodies, showed a distinct band at 55 kDa (Figure 1A), which was the expected molecular weight of the glycosylated monomer. The two biologically active modules of properdin TSR4 and TSR5 were expressed together in tandem as previously described, fused to MBP (7), and is also shown on an SDS-PAGE gel, which has a molecular weight of 55 kDa (Figure 1B). Using gel filtration chromatography, we found that human properdin eluted as a mixture of monomer, dimer and trimer; a negligible amount probably formed aggregates. Nearly 60% of MBP-TSR4+5 appeared as a monomer while nearly 40% was found to migrate as a dimer (data not shown).

**Figure 2:**
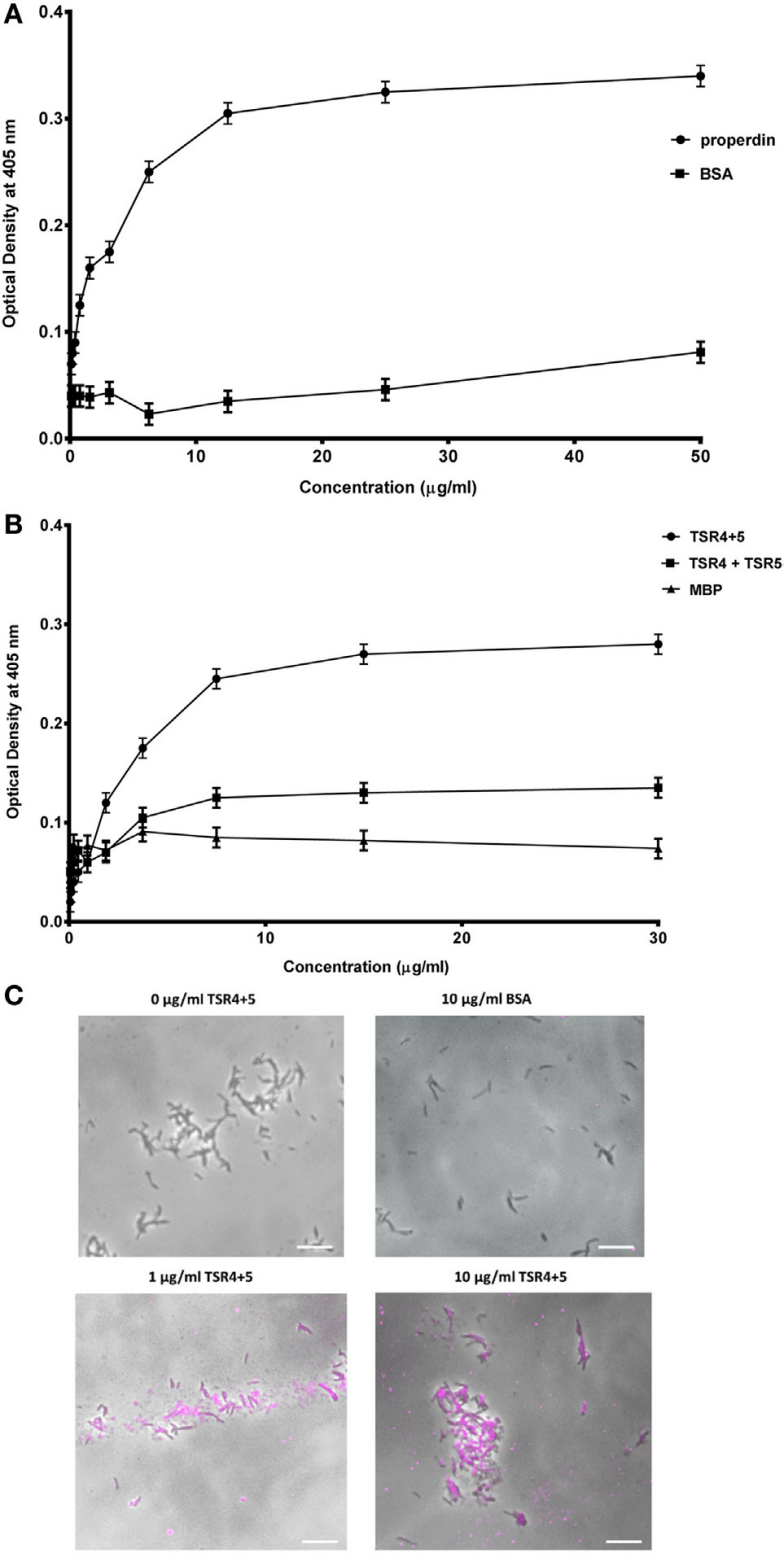
Human properdin binds mycobacteria *via* thrombospondin repeats (TSR) 4+5. **(A)** Properdin binding to mycobacteria; BSA was used as a negative control protein. **(B)** Comparison between TSR4+5 and individual TSR4 and TSR5 binding to mycobacteria; maltose-binding protein (MBP) as negative control. Assays were conducted in 10 mM HEPES, 140 mM NaCl, 0.5 mM CaCl_2_ + 0.5 mM MgCl_2_, 100 µg/ml hen ovalbumin, and pH 7.5. Serial dilutions of properdin were incubated in mycobacteria coated wells followed by incubation with mouse anti-properdin monoclonal antibody and mouse anti-BSA monoclonal antibody, respectively; serial dilutions of TSR4+5, TSR4 or TSR5 were incubated in another set of mycobacteria coated wells followed by incubation with mouse anti-MBP monoclonal antibody. Anti-mouse IgG conjugated with alkaline phosphatase and substrate *p*-nitrophenol phosphate were incubated in both sets of wells, and the color was measured at 405 nm using a plate reader. Assay was conducted in quadruplicate. Error bars represent SD. **(C)** Differential direct binding of 0, 1, and 10 µg/ml of TSR4+5 to *Mycobacterium bovis* BCG. 10 µg/ml of BSA was used as a negative control. Cells were incubated for 2 h with either TSR4+5 or BSA. Cells were washed, fixed, and stained with mouse anti-MBP monoclonal antibody followed by goat anti-mouse 1gG-conjugated with AlexaFluor488. Images are shown as single sections taken using a Leica DM4000 microscope; bar scale 10 µm.

The binding of properdin to *M. bovis* BCG was observed to be in a dose-dependent manner; BSA was used as a negative control that showed almost no binding (Figure 2A). TSR4+5 binding was also observed to be in a dose-dependent manner. MBP was used as a negative control (Figure 2B). The two binding curves cannot be compared quantitatively, as different detection antibodies were used. Because the MBP-TSR4+5 recombinant protein and properdin monomer have about the same molecular weight, 5 µg of TSR4+5 corresponds in molar terms to about 5 µg of properdin monomer (Figures 1A,B). The binding of a mixture of the two separately expressed TSR4, and TSR5 is much lower than that of the combined expressed TSR4+5 (Figure 2B). For this comparison, the same detection antibody was used. These results suggest that both TSR4 and TSR5 modules contribute to the interaction with *M. bovis* BCG, and that TSR4+5 binds with similar characteristics to that of whole properdin on *M. bovis* BCG surface. These results were further confirmed using microscopy where TSR4+5 specifically bound to *M. bovis* BCG in a dose-dependent manner (Figure 2C).

### Properdin Inhibits Uptake of *M. bovis* BCG by THP-1 Cells

**Figure 3:**
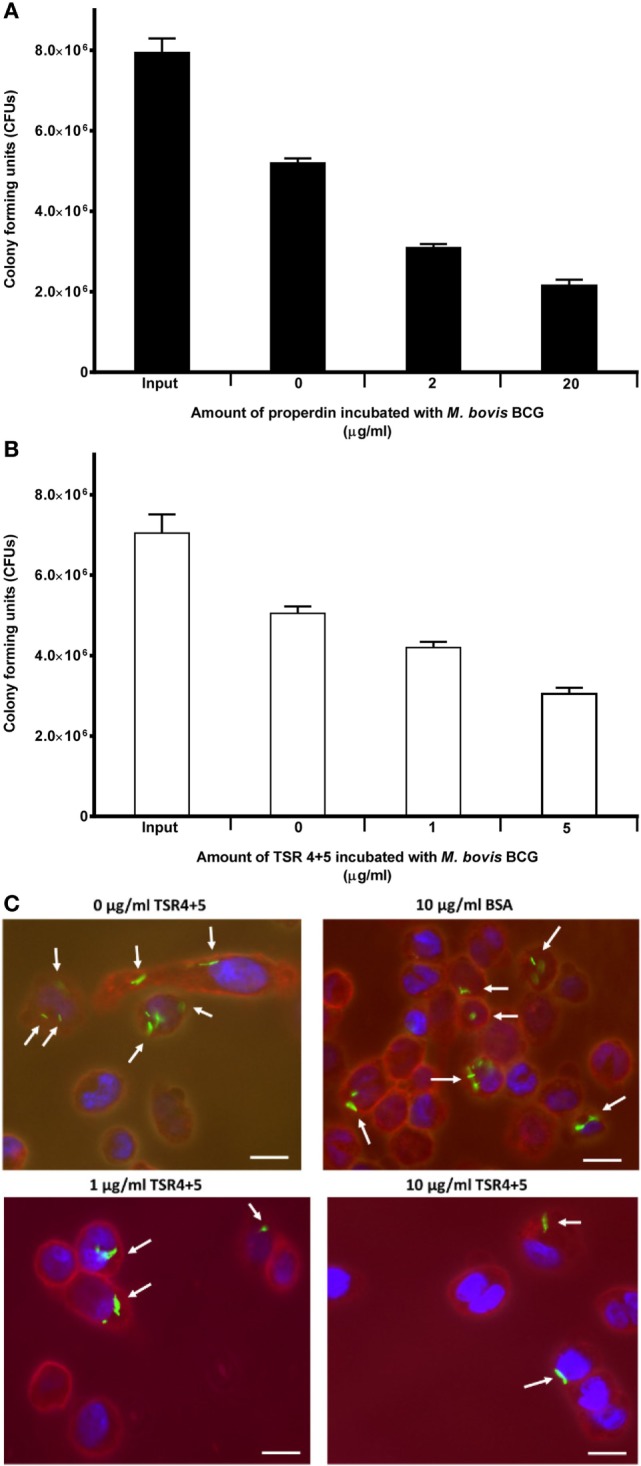
Effect of properdin and thrombospondin repeats (TSR) 4+5 on the phagocytosis of *Mycobacterium bovis* BCG by THP-1 cells. **(A)** *M. bovis* BCG was treated with properdin at concentrations of 0, 2, and 20 µg/ml or with **(B)** TSR4+5 at concentrations 0, 1, and 5 µg/ml. The mycobacteria were incubated with macrophage for 2 h. After THP-1 cell lysis, surviving internalized *M. bovis* BCG were measured by plating lysates on 7H10 media to obtain colony-forming units (CFUs). The input value is the starting number of *M. bovis* BCG added to the THP-1 cells, before phagocytosis. A one-way ANOVA test was performed on the data to determine significant differences in CFU count by properdin or TSR4+5. All comparisons were significant (*p* < 0.05), unless where shown (ns, not significant, *p* > 0.05). Samples were analyzed in triplicate. **(C)** Differential uptake of GFP-*M. bovis* BCG by THP-1 macrophages after treatment with 0, 1, and 10 µg/ml of TSR4+5, or 10 µg/ml of BSA, used as a negative control. Cells were incubated for 2 h. Cells were then washed, fixed, and stained with AlexaFluor546-conjugated wheat germ agglutinin to reveal the plasma membrane (red), and the nucleus was stained with DAPI (blue). Images are shown as single sections, taken using a Leica DM4000 microscope; bar scale 10 µm.

Properdin inhibited the uptake of *M. bovis* BCG by THP-1 cells. At a concentration of 20 µg/ml, uptake of *M. bovis* BCG was significantly reduced by properdin (Figure 3A). TSR4+5 was also able to substantially inhibit uptake of *M. bovis* BCG by THP-1 cells (Figure 3B). The effect of properdin and TSR4+5 on the phagocytosis of *M. bovis* BCG was dose dependent. The input number of *M. bovis* BCG was about 7.8 × 10^6^ CFU/ml, which was the total number of bacteria added to THP-1 cells. Without properdin or TSR4+5, 5.0 × 10^6^ CFU/ml of *M. bovis* BCG was phagocytosed which was approximately 66% efficiency of phagocytosis compared with the input number. At the highest concentration tested, properdin showed an inhibition of uptake of approximately 60% compared with *M. bovis* BCG with no properdin (Figure 3A). For TSR4+5, the effect on *M. bovis* BCG was slightly lower at approximately 40% inhibition (Figure 3B). These results were also confirmed by microscopy, with TSR4+5 having a suppressive effect on the uptake of GFP-expressing *M. bovis* BCG by THP-1 cells (Figure 3C). PMA stimulation was used to induce differentiation of THP-1 cells before incubation with *M. bovis* BCG. PMA has been shown to activate protein kinase C and increase cell adherence and expression of surface markers associated with macrophage differentiation (30). These data show that (i) properdin has an anti-opsonic effect on *M. bovis* BCG, inhibiting phagocytosis; and (ii) TSR4+5 modules play a major role in this interaction of *M. bovis* BCG and macrophages. These observations demonstrate, for the first time, a novel, non-complement-related role for properdin in host–pathogen interactions in tuberculosis.

### Properdin Induces a Pro-Inflammatory Response During the Early Phase of Phagocytosis of *M. bovis* BCG by THP-1 Cells

**Figure 4:**
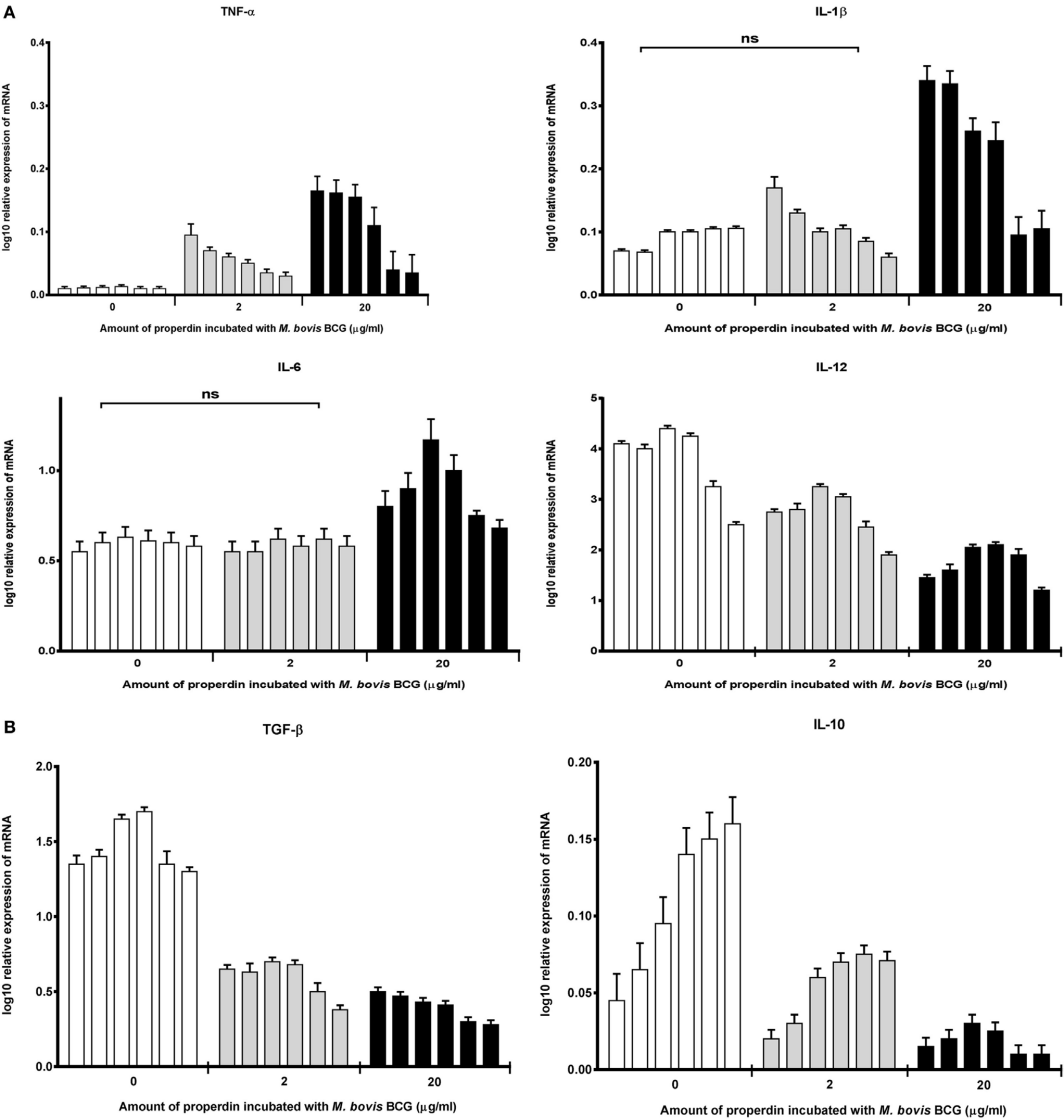
Temporal mRNA expression profile of cytokines produced by THP-1 cells incubated with different concentrations of properdin and *Mycobacterium bovis* BCG. **(A)** Pro-inflammatory cytokines: TNF-α, IL-1β, IL-6, and IL-12; **(B)** anti-inflammatory cytokines: TGF-β and IL-10. The expression of each cytokine was measured using qPCR, and the relative expression [relative quantification (RQ)] calculated by normalizing the data using human 18S rRNA expression as a control. The RQ value was calculated using the formula: RQ = 2^−ΔΔCt^. Assays were conducted twice in triplicates. Error bars represent SD. A two-way ANOVA test was performed on the data to determine significant differences in expression of cytokine production by properdin. All comparisons were significant (*p* < 0.05), unless where shown (ns, not significant, *p* > 0.05).

The effect of properdin on the inflammatory response during the phagocytosis of *M. bovis* BCG was measured. The gene expression of key pro- and anti-inflammatory cytokines in tuberculosis was determined using quantitative real-time PCR. Our data showed that properdin significantly enhanced the upregulation of pro-inflammatory cytokines TNF-α, IL-1β, and IL-6 from THP-1 cells challenged by *M. bovis* BCG (Figure 4A), particularly at the initial stage of uptake (within the first hour of phagocytosis), which decreased gradually toward the later stages of phagocytosis. The increase in TNF-α transcript was particularly striking as TNF-α is well known for activating macrophages for killing of intracellular mycobacteria. In addition, TNF-α is a key mediator in the early stages of granuloma formation. By contrast, the anti-inflammatory cytokines measured from THP-1 cells (IL-10 and TGF-β) were shown to be downregulated in the presence of properdin, when challenged by *M. bovis* BCG (Figure 4B). IL-12 also appeared to be downregulated (Figure 4A). Properdin, therefore, appears to play an important role in pro-inflammatory cytokine production by macrophages infected by *M. bovis* BCG, which may have significant implications in shaping the adaptive immune response during *M. tuberculosis* infection.

**Figure 5:**
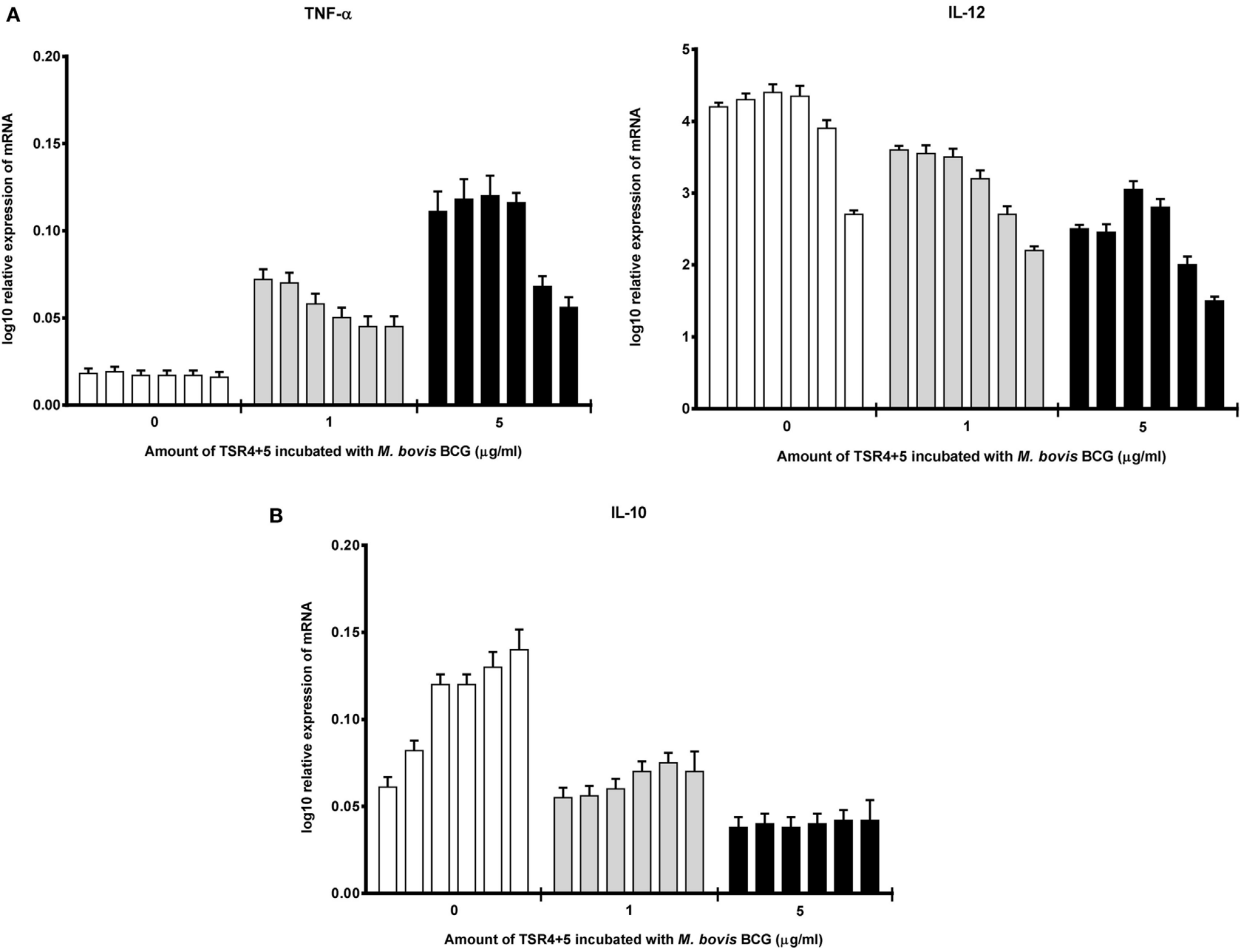
Temporal mRNA expression profile of cytokines produced by THP-1 cells incubated with different concentrations of thrombospondin repeats (TSR) 4+5 and *Mycobacterium bovis* BCG. **(A)** Pro-inflammatory cytokines: TNF-α and IL-12; **(B)** anti-inflammatory cytokine: IL-10. The expression of each cytokine was measured using qPCR, and the relative expression [relative quantification (RQ)] calculated by normalizing the data using human 18S rRNA expression as a control. The RQ value was calculated using the formula: RQ = 2^−ΔΔCt^. Assays were conducted twice in triplicates. Error bars represent SD. A two-way ANOVA test was performed on the data to determine significant differences in expression of cytokine production by TSR4+5. All comparisons were significant (*p* < 0.05), unless where shown (ns, not significant, *p* > 0.05).

Cytokine gene expression by THP-1 cells infected with *M. bovis* BCG were also studied in the presence of TSR4+5, which revealed that TSR4+5 also has a significant effect on the pro-inflammatory response. TNF-α was upregulated (Figure 5A), while IL-10 was found to be downregulated (Figure 5B), during the first hour of phagocytosis. IL-12 was also shown to be significantly downregulated (Figure 5A). These data mirror the observations for properdin, and hence, validate the importance of TSR4+5 in the binding of properdin to *M. bovis* BCG and in its modulation of the inflammatory response. These data are also similar to recent published observations of another complement regulatory protein, factor H (19), thus offering potentially novel insights into the involvement of these proteins in host–pathogen interactions in tuberculosis.

### Multiplex Analysis of Cytokine Secretion

**Figure 6:**
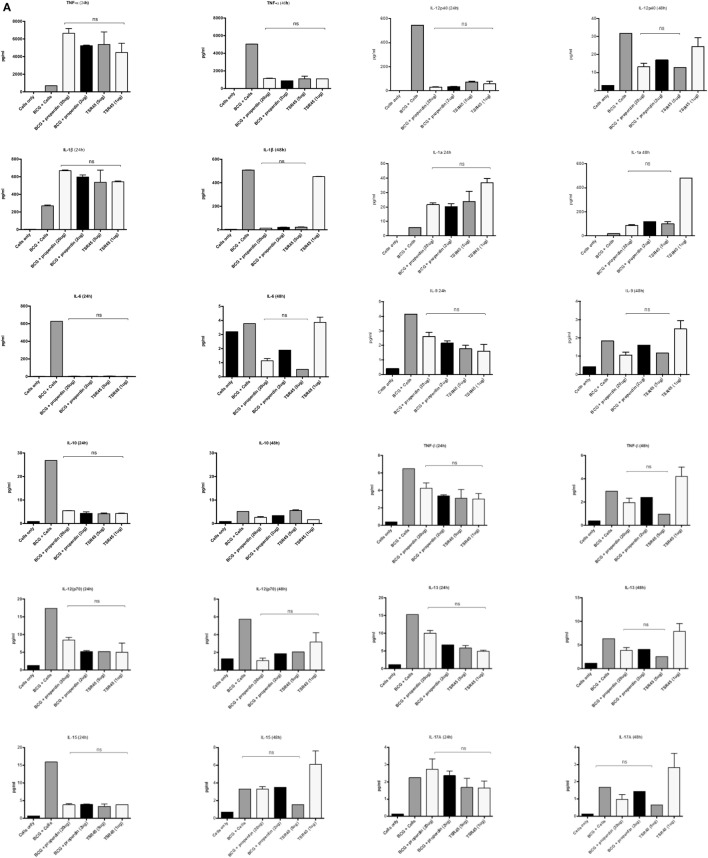

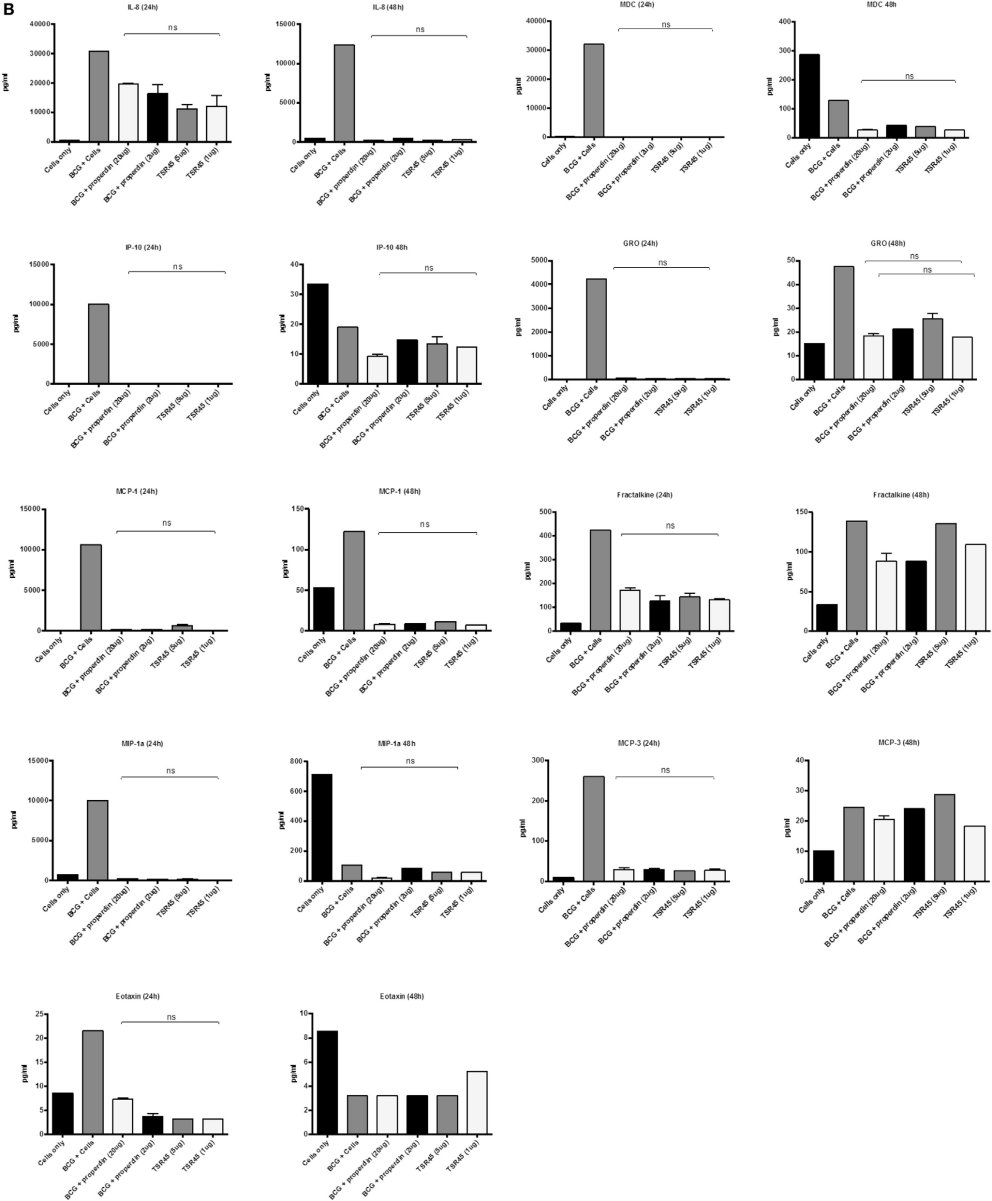

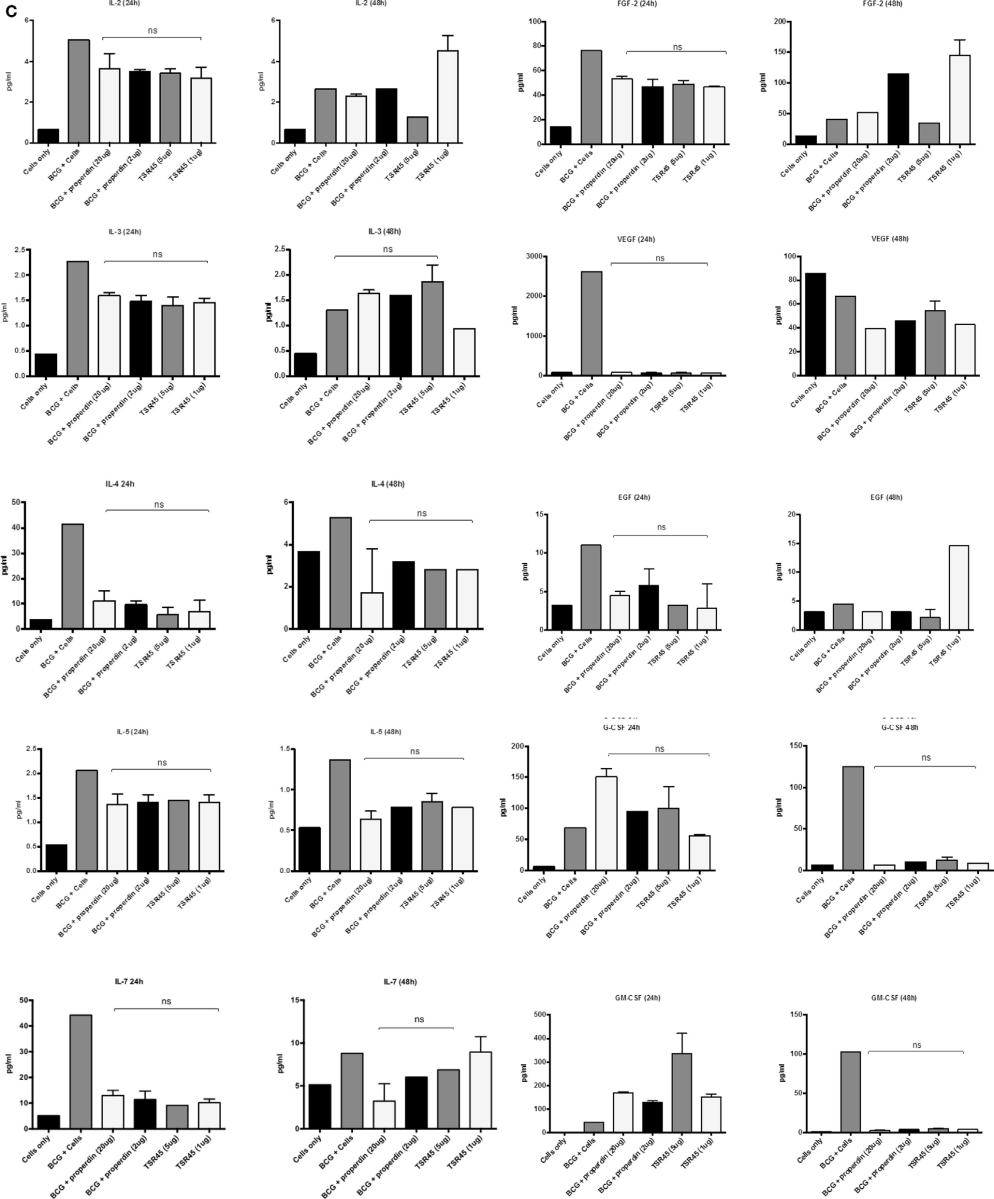

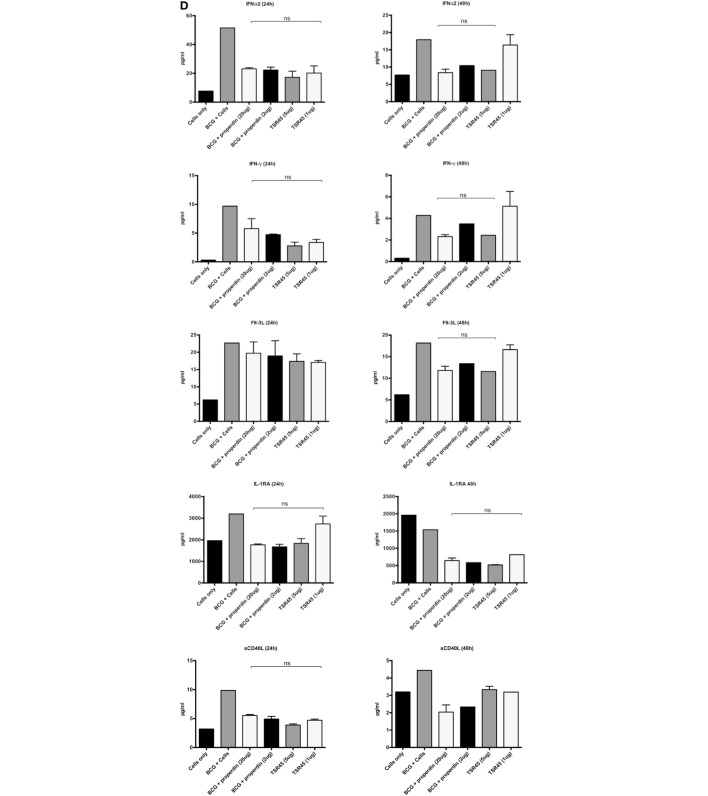
Multiplex cytokine analysis of supernatants collected at 24 and 48 h phagocytosis of *Mycobacterium bovis* BCG by THP-1 cells incubated with or without properdin and thrombospondin repeats (TSR) 4+5. The supernatants were collected from phagocytosis assay of *M. bovis* BCG in the presence or absence of properdin and TSR4+5 at 24 and 48 h time point. The levels of cytokine production were measured for **(A)** (IL-6, IL-10, IL-12p40, IL-12p70, IL-1α, IL-1β, TNF-α, IL-13, IL-15, IL-17A, IL-9, and TNF-β), **(B)** chemokines (MCP-3, MDC, Eotaxin, Fractalkine, GRO, IL-8, IP-10, MCP-1, and MIP-1α), **(C)** growth factors (IL-9, IL-2, EGF, FGF-2, G-CSF, GM-CSF, IL-3, IL-4, IL-5, IL-7, and VEGF), and **(D)** related ligands and receptors (IFN-α2, IFN-γ, FLT-3L, IL-1RA, and sCD40L) using multiplex analysis. Error bars represent SD. A one-way ANOVA test was performed on the data to determine significant differences in expression of cytokine production by properdin or TSR4+5. All comparisons were significant (*p* < 0.05), unless where shown (ns, not significant, *p* > 0.05). Supernatants were analyzed in duplicate.

The inflammatory response during the phagocytosis of *M. bovis* BCG by THP-1 cells was further determined by measuring the secretion of cytokines, chemokines, and other growth factors using the Multiplex analysis of supernatants collected at 24 and 48 h post phagocytosis (Figures 6A–D). The secretion of pro-inflammatory cytokines TNF-α, IL-1β, and IL-1α was significantly enhanced by treatment with properdin or TSR4+5 at the 24 h time point (Figure 6A). The enhancement of these pro-inflammatory cytokines can be critical for controlling mycobacterial infection, particularly in the formation of the granuloma. However, by 48 h, there was a decrease in the production of pro-inflammatory cytokines (IL-6, IL-12p40, IL-12p70, IL-1α, IL-1β, TNF-α, IL-13, IL-15, and IL-9) in the presence of properdin- and TSR4+5-treated *M. bovis* BCG (Figure 6A). Properdin and TSR4+5 also downregulated the anti-inflammatory response such as IL-10 after 24 and 48 h of phagocytosis, although this was less pronounced for IL-12 at 48 h (Figure 6A). These observations again mirror the initial responses observed in cytokine gene expression of during the first few hours of phagocytosis, in the presence of properdin or TSR4+5 (Figures 5A,B). The effect of properdin and TSR4+5 also led to marked downregulation of a number of growth factors MCP-3 (24 h), MDC, Eotaxin, Fractalkine (24 h), GRO (24 h), IP-10, MCP-1, MIP-1, VEGF, G-CSF (48 h), GM-CSF (48 h), and VEGF (24 h) (Figures 6B,C). Additional ligands and receptors (IFN-α2, IFN-γ, FLT-3L, IL-1RA, and sCD40L) did not show any significant changes (Figure 6D).

## Discussion

We have previously shown that a complement regulatory protein, factor H, can bind to *M. bovis* BCG and inhibit its uptake by THP-1 macrophages (19). Factor H can also enhance the pro-inflammatory response during this host–pathogen interaction (19). This study highlighted a novel complement-independent property of factor H as an anti-opsonin and in the modulation of the inflammatory response against a pathogen. With the goal of further elucidating the role of complement control proteins in the early stages of mycobacterial infection, this study looked at the role of properdin, an upregulator of the alternative complement pathway. Properdin and thrombospondin repeat (TSR) modules TSR4+5 were shown to bind to *M. bovis* BCG and inhibit bacterial uptake by THP-1 cells, augmenting the inflammatory response. These observations are similar to what has been observed previously with factor H (19), which is intriguing, since properdin and factor H have opposing effects on the regulation of complement activation (3). These findings were also consistent with previous reports, which have demonstrated that properdin deficient mice have a reduced M1 (IL-1β) and increased M2 (arginase-1, MCP-1, IL-10) profile, crucial for the tumor microenvironment (31). This suggests that the production of IL-1β and reduction in IL-10 mediated by properdin may be required for protection against *M. tuberculosis* in the initial phase of infection.

The functions of properdin have been extensively investigated within the remit of the complement alternative pathway, and its involvement with pathogens has largely been characterized as complement dependent. In this study, we aimed to look at the complement-independent interaction of properdin with mycobacteria (i.e., effects in the absence of other complement proteins), with a view to examining its possible role in the pathogenesis of tuberculosis. The role of complement in tuberculosis has been examined, but little is understood about the role of the individual complement proteins in tuberculosis infection, especially complement control proteins. Properdin has been shown to play a role in a number of pathogenic infections such as those by *C. pneumoniae*, in which properdin promotes complement C3b deposition and opsonization (12). A recent study also demonstrated that a low dose of properdin, which is highly polymerized, is able to protect against *N. meningitidis* and *Streptococcus pneumoniae*, by assembling the alternative complement pathway (16). The central premise in these recent studies is that properdin is an upregulator of the complement alternative pathway, and thus, when in contact with pathogens, the alternative pathway is triggered and stabilized by properdin.

Properdin has also been shown to enhance the uptake of apoptotic T cells by dendritic cells (DCs) and macrophages, thus promoting phagocytosis (27). Properdin may also bind to the DNA found to be exposed on apoptotic and necrotic cells, suggesting that this may also be a crucial site for alternative pathway activation (32). There is recent evidence to suggest that properdin, locally produced by tolerogenic DCs, binds to necrotic cells, confirming its role a pattern recognition molecule of the innate immunity. In addition, properdin is also involved in the interaction of DC and T cell responses. Interestingly, silencing of properdin by treating DCs with siRNA in the presence/absence of IFN-γ reduced the proliferation of allogenic T cells (33). Properdin binds to early apoptotic cells via sulfated GAGs, resulting in C3b deposition and uptake by phagocytes. Activation of neutrophils drives the deposition of properdin, which binds apoptotic T cells. Since properdin has been shown to bind apoptotic cells via GAGs (27) or DNA, it remains unclear what other ligands and receptors are involved in properdin–apoptotic cell interaction. It is likely that properdin as a soluble factor is acting as an adaptor molecule. Furthermore, properdin also binds to NKp46 expressed on natural killer cells, innate lymphoid cells (ILC)1 and ILC3. This study demonstrated that the control of meningococcal infection was dependent on NKp46 and group 1 ILCs, further elucidating the role of properdin as an independent pattern recognition molecule (34).

In our study, we show that purified native properdin and TSR4+5 bind to *M. bovis* BCG in a dose-dependent manner, suggesting that the binding of properdin to *M. bovis* BCG may be *via* TSR4+5. The physiological concentration of properdin in serum is about 25 µg/ml (35). We also demonstrate that coating of *M. bovis* BCG with properdin inhibits the uptake of the bacterium by THP-1 cells; however, only about 60% inhibition was achieved at the highest dose of native properdin. TSR4+5 was also able to mirror the effects of properdin, in inhibiting the uptake of *M. bovis* BCG by macrophages by up to 40%.

The recruitment of properdin by mycobacteria may be particularly crucial in the initial stages of tuberculosis infection, when after inhalation, the first host cell *M. tuberculosis* encounters is the alveolar macrophage.

In the lungs, mycobacteria are phagocytosed by alveolar macrophages, which are unable to completely eliminate them, and so produce crucial chemoattractants (36), which recruit inflammatory cells such as neutrophils, macrophages, γδ-T cells, and natural killer cells that stimulate inflammation and tissue remodeling (36–38). Our findings may be indicative of the early inflammatory processes *in vivo*, involved in granuloma formation, which are nodular-type lesions that cordon-off *M. tuberculosis* infection, and provide an environment for the bacilli to persist and survive as a latent infection. TNF-α and IFN-γ are involved in recruitment of cells in the granuloma (39, 40). Thus, properdin may play a role in granuloma formation by promoting pro-inflammatory cytokines. Inflammatory balance is essential particularly of Th2/Th1 cytokines, which are required to maintain a protective granuloma (41). This is determined by the balance in IFN-γ/TNF-α versus IL-4/IL-10/TGF-β within the granuloma. Properdin and TSR4+5 may be implicated in maintaining this balance. It is not known whether complement proteins reside in the granuloma; however, during infection, complement proteins may be produced locally at sites of infection. Properdin may be secreted by neutrophils, monocytes and T cells locally at the site of infection (3). Thus, innate immune molecules residing in or being recruited at sites of infection may play a role in the balance of Th1/Th2, which may cause granuloma necrosis and replication of *M. tuberculosis* (41–43).

TNF-α was dramatically increased in the first 24 h of phagocytosis in the presence of properdin and TSR4+5, compared to non-treated mycobacteria. TNF-α plays a major role in granuloma formation and our results suggest that properdin may have a role in potentiating the pro-inflammatory response that results in granuloma formation. These observations are further strengthened by the concurrent increase in IL-1α levels over 24 h which have been shown to be key in macrophage proliferation and maturation during granuloma formation (44).

During phagocytosis, pro- and anti-inflammatory cytokines were produced by THP-1 cells when treated with properdin or TSR4+5. In the initial stages of infection by *M. bovis* BCG, pretreated with properdin, during phagocytosis, the expression of TNF-α was significantly enhanced. Other pro-inflammatory responses that were also elevated in the presence of properdin at initial stages of infection are IL-1β and IL-6. IL-1β is a mediator of inflammation and is required for host resistance to *M. tuberculosis* infection (45). IL-6 is a biomarker for tuberculosis, as increased levels are observed in patients with tuberculosis (46) that is required for a T cell response against *M. tuberculosis* infection (47, 48). Conversely, IL-10, IL-12, and TGF-β were downregulated by properdin, thus suppressing the anti-inflammatory response. The downregulation of IL-12 by properdin *in vivo* may suppress the Th1 response. TSR4+5 was also able to mimic the cytokine response like properdin, suggesting that the modules responsible for the major part of the interaction with *M. bovis* BCG may be TSR4+5.

Macrophages play a significant role in the innate immune response to pathogens and so are also crucial for an adaptive immune response (49). However, *M. tuberculosis* can evade the innate immune defense, inhibiting phagosome maturation (36), resisting anti-microbial agents damaging the bacterial cell wall and facilitating replication within the host and escaping early immune recognition. Thus, these pathogens interfere with the early immune response and the induction of pro-inflammatory cytokines (49).

IL-10 and TGF-β suppression by properdin may enhance the clearance of mycobacteria by the host during the early stages of *M. tuberculosis* infection. After phagocystosis of *M. tuberculosis*, IL-10 has been shown to block phagolysosome maturation and antigen presentation by macrophages, thus aiding the survival of the pathogen (50, 51). Furthermore, IL-10 can inhibit the generation of reactive oxygen and nitrogen intermediates in IFN-γ activated macrophages, which are required for intracellular killing (52, 53). The enhanced levels of IL-10 and TGF-β in the lungs of active tuberculosis patients demonstrate a weakened immune response to *M. tuberculosis*, and hence, a role in the pathogenesis and disease progression (54, 55). VEGF was also found to be at a significantly higher level in tuberculosis patients with extrapulmonary tuberculosis (EPTB) than those with pulmonary disease (56). In our study, properdin and TSR4+5 seems to result in a marked elevation of VEGF after 48 h. Since our data also shows that mycobacteria have a reduced phagocytosis by macrophages, the resulting extracellular bacteria may be encouraged by VEGF to disseminate. The beneficial effect of properdin may be to inhibit the mechanisms involved in evasion and, thus, facilitate a protective response against mycobacterial infection.

The downregulation of IL-12 by properdin or TSR4+5 may be due to the reduced phagocytosis of *M. bovis* BCG, thus, downregulating the Th1 response. This may be necessary for the Th1/Th2 homeostasis in the protective granuloma (41, 57, 58). Both IL-10 and TGF-β levels were supressed, whilst TNF-α was elevated during the first 24 h after phagocytosis.

Although *M. bovis* BCG shares 99% genome homology to *M. tuberculosis*, there are some genetic differences which lead to its avirulence. The major difference between *M. bovis* BCG and *M. tuberculosis* is the large genomic deletion RD1, which causes the loss of various virulent genes coding for proteins such as ESAT-6, CFP-10 and also a bacterial secretion system (59, 60). Therefore the findings in our study will need to be validated using virulent strains of *M. tuberculosis*.

Properdin deficiency renders the host susceptible to a range of bacterial infections, especially *Neisseria* species. Three types of properdin deficiency have been reported: type I (absence of the properdin protein), type 2 (low level of properdin about 1–10% found in the serum), and type 3 deficiency (normal levels of protein being produced, but functionally defective). The most commonly reported deficiency is the type I properdin deficiency that exhibits fulminant infections. The incidence of tuberculosis has not been reported in properdin deficient subjects, possibly due to the majority of studies being in Scandinavia or western Europe, in populations where there is a low incidence of tuberculosis.

The data in this study suggest that properdin, *via* TSR4+5, may help in the clearance of mycobacterial infection by circumventing pathogen immune evasion strategies by upregulating the pro-inflammatory response. Properdin may also promote the formation and maintenance of the protective granuloma. The data in this study give further insights into the involvement of complement regulatory proteins in shaping the cellular immune response against mycobacteria in a complement activation-independent manner. Further studies are needed to fully characterize the nature and extent of involvement of properdin in tuberculosis pathogenesis, particularly in the early stages of infection.

The complement-independent interaction between human properdin and mycobacteria is a novel observation, which is independent of C3b deposition and aggregation of properdin (61). This is consistent with our recent study where we have shown that properdin can recognize chemical patterns on nanoparticles *via* TSR4+5 and modulate immune response by THP-1 cells (26) without involving complement activation/deposition. In conclusion, properdin may be involved in modulating host-pathogen interactions in tuberculosis. However, further studies are needed on pathogenic *M. tuberculosis* and *in vivo*, to understand the precise role of this complement regulatory protein in pathogenesis, which may give new insights into therapies against this formidable disease.

## Author Contributions

MA-M, AT, MA-A, and LK carried out crucial experiments. SA, MNA-A, AAP, VM, EMG, AK, and RBS provided crucial reagents and expertise. UK, AT and LK wrote the manuscript in addition to designing the experiments.

## Conflict of Interest Statement

The authors declare that the research was conducted in the absence of any commercial or financial relationships that could be construed as a potential conflict of interest.

## Footnotes

^1^http://imagej.nih.gov/ij.

^2^http://blast.ncbi.nlm.nih.gov/Blast.cgi.
